# Acoustic diagnosis of pulmonary hypertension: automated speech- recognition-inspired classification algorithm outperforms physicians

**DOI:** 10.1038/srep33182

**Published:** 2016-09-09

**Authors:** Tarek Kaddoura, Karunakar Vadlamudi, Shine Kumar, Prashant Bobhate, Long Guo, Shreepal Jain, Mohamed Elgendi, James Y Coe, Daniel Kim, Dylan Taylor, Wayne Tymchak, Dale Schuurmans, Roger J. Zemp, Ian Adatia

**Affiliations:** 1 Department of Electrical and Computer Engineering, University of Alberta, Edmonton, Canada; 2 Pediatric Pulmonary Hypertension Service, Pediatric Cardiac Critical Care Unit, Stollery Children’s Hospital, Mazankowski Alberta Heart Institute, University of Alberta, Edmonton, Canada; 3 Department Computing Science, University of Alberta, Edmonton, Canada; 4 Department of Medicine, Division of Cardiology, Cardiac Catheterization Laboratories, University of Alberta Hospital, Mazankowski Alberta Heart Institute, Edmonton, Canada

## Abstract

We hypothesized that an automated speech- recognition-inspired classification algorithm could differentiate between the heart sounds in subjects with and without pulmonary hypertension (PH) and outperform physicians. Heart sounds, electrocardiograms, and mean pulmonary artery pressures (mPAp) were recorded simultaneously. Heart sound recordings were digitized to train and test speech-recognition-inspired classification algorithms. We used mel-frequency cepstral coefficients to extract features from the heart sounds. Gaussian-mixture models classified the features as PH (mPAp ≥ 25 mmHg) or normal (mPAp < 25 mmHg). Physicians blinded to patient data listened to the same heart sound recordings and attempted a diagnosis. We studied 164 subjects: 86 with mPAp ≥ 25 mmHg (mPAp 41 ± 12 mmHg) and 78 with mPAp < 25 mmHg (mPAp 17 ± 5 mmHg) (p  < 0.005). The correct diagnostic rate of the automated speech-recognition-inspired algorithm was 74% compared to 56% by physicians (p = 0.005). The false positive rate for the algorithm was 34% versus 50% (p = 0.04) for clinicians. The false negative rate for the algorithm was 23% and 68% (p = 0.0002) for physicians. We developed an automated speech-recognition-inspired classification algorithm for the acoustic diagnosis of PH that outperforms physicians that could be used to screen for PH and encourage earlier specialist referral.

Pulmonary hypertensive vascular disease is a serious condition that imposes a global disease burden in both privileged and under privileged communities^1^. If untreated pulmonary hypertensive vascular disease has a high mortality whether the cause is idiopathic pulmonary arterial hypertension, genetic mutation or a complication of congenital, acquired or autoimmune cardiac or pulmonary disease^2^. Pulmonary hypertension may be diagnosed late because early clinical recognition is difficult even after the onset of symptoms or because the associated illness confounds the clinical picture. Patients with idiopathic pulmonary arterial hypertension often suffer substantial delay from onset of symptoms to diagnosis despite contact with medical services^3^. There has been a call for earlier detection with improved screening because late diagnoses of patients who are symptomatic in an advanced WHO functional class have a poor outcome. This need has been highlighted in reviews of multiple large national PH registries^4^,^5^. There is a general consensus that early treatment with combination therapy may improve outcome^6^,^7^. There is, therefore, a pressing and unmet need to explore diagnostic screening methods that are cost effective, accurate, non-invasive and result in a timely clinical diagnosis of pulmonary hypertension.

Pulmonary hypertension may be diagnosed by auscultation, which emphasizes the loudness of the pulmonary component of the second heart sound and the width of splitting between the aortic and pulmonary components. However, precise automated localization of the second heart sound and measurement of the splitting interval between the components of the second heart sound have proved difficult. We have taken an alternative approach and investigated the diagnosis of PH by harnessing electronic stethoscope recordings with advanced signal processing and the application of speech and language recognition algorithms^8^,^9^,^10^. Automated speech-recognition-inspired-classification algorithms simultaneously take into account many aspects of a heart sound signal, including the shape, frequency components, and amplitude. Therefore, we sought to develop a method to diagnose pulmonary hypertension from digital recordings of heart sounds using automated-machine-learning and speech-recognition-inspired –classification algorithms. In addition, we investigated how the developed algorithm would perform in comparison to clinicians trained in auscultation.

## Methods

### Ethics Approval

The Research Ethics Board of the University of Alberta approved the study. All subjects or their parents gave informed and written consent to participate in the study. Informed assent was obtained from children who had reached sufficient developmental ability. The research was conducted according to the principles of the Declaration of Helsinki.

### Clinical Data Collection

We approached all subjects undergoing a right heart catheterization that was required for management of their underlying condition for inclusion in the study. We excluded subjects with congenitally abnormal aortic, pulmonary and prosthetic valves. We defined PH as a mean PAp ≥ 25 mmHg according to internationally accepted definitions in adults and children^11^,^12^,^13^,^14^.

Heart sounds were recorded simultaneously with direct pulmonary artery pressure measurements and electrocardiogram (EKG) obtained during heart catheterization performed in a standard manner. Other hemodynamic data including, heart rate, pulmonary artery wedge pressure (PAWp) or left atrial pressure (LAp), right atrial pressure (RAp), oxygen consumption, systemic pressure and estimates of systemic and pulmonary blood flow were collected within 5–10 minutes of the acoustic recordings. In subjects with cardiac shunts systemic and pulmonary blood flow indexed to body surface area was estimated using the Fick equation. Oxygen consumption was measured by mass spectroscopy using the Amis 2000 or the Innocor (Innovision, Denmark)^15^. In subjects without cardiac shunts we used Swan-Ganz thermodilution catheters to estimate cardiac output using room temperature saline and the average of 3 readings that differed by less than 10%. We calculated the pulmonary (PVRI), systemic vascular resistance index (SVRI), diastolic pressure gradient and pulmonary artery capacitance index using standard formula.

Heart sounds were recorded for 20 seconds using the Zargis Signal X system (Princeton NJ) using recordings obtained simultaneously from the traditional auscultation areas for aortic (2^nd^ right intercostal space), pulmonary (2^nd^ left intercostal space), tricuspid (4^th^ right intercostal space), and cardiac apex. The Zargis Signal X system is a method that we adapted to simultaneously record the heart sounds together with an ECG and the pulmonary artery pressure tracings. Four acoustic sensors or microphones with a diaphragm were placed with adhesive on to the subject’s chest to record the heart sounds. The sound signal was amplified and displayed as a phonocardiogram tracing which could be saved, exported to MATLAB and analyzed later. For this study we used only the recordings from the 2^nd^ left intercostal space and the ECG tracing. The pulmonary artery pressure tracing was used to define the pulmonary arterial pressure at the time of the heart sound recording.

### Identification of a region of interest containing S2

**Figure 1:**
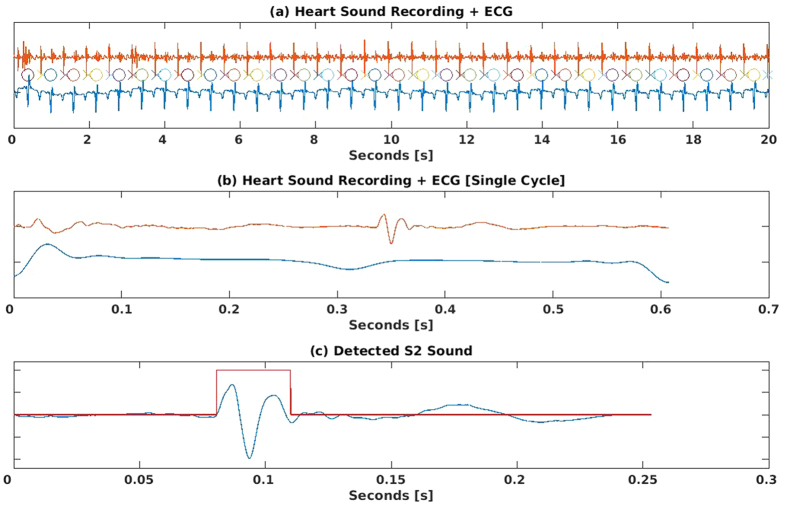
(**a**–**c**) illustrate how we identified an area of interest that included the second heart sound (S2) using automated detection of the R and T waves in the ECG to an area of approximately 30% of the cardiac cycle on the phonocardiogram. The x –axis shows time in seconds and the y-axis is the relative amplitude of the signals. Figure 1a. Simultaneous phonocardiographic and electrocardiographic tracings. We illustrate simultaneous phonocardiographic and electrocardiographic tracings with an example of a 20 second recording of the phonocardiogram (top tracing) and electrocardiogram (lower tracing) in a patient with normal pulmonary artery pressures (mean PA pressure <25 mmHg). Automatically detected R-waves of the QRS complex are marked with an O, and T-waves are marked with an X. The second heart sound (S2) window is identified in the algorithm as 30% of the cardiac cycle around the T wave. Figure 1b. Simultaneous phonocardiographic and electrocardiographic tracings of a single cardiac cycle. The phonocardiographic and electrocardiographic tracing from a single cardiac cycle from the same subject and recording in Figure 1a,c. Identifying the window that included S2 on the phonocardiogram. There is a 0.25 second window around the T wave, which, identified the area of interest, used in the algorithm. The loudest signal in the boxed area was designated the second heart (S2) shown in the phonocardiogram at approximately 0.1 seconds on the x-axis.

**Figure 2:**
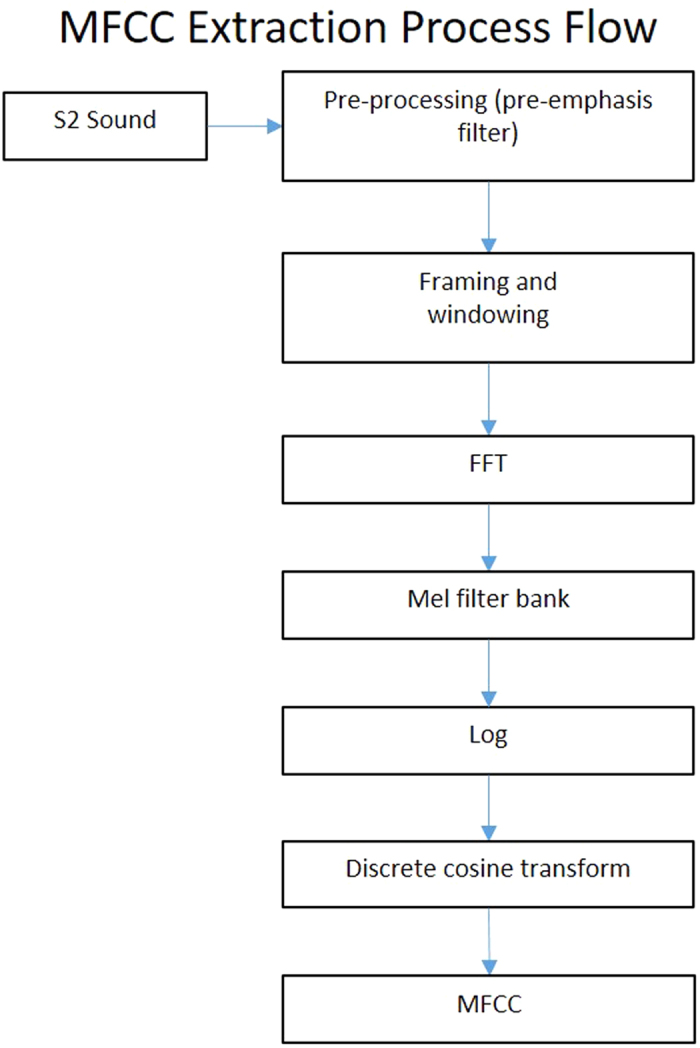
Flow diagram of MFCC extraction process. This flow diagram depicts the process for extraction of Mel-Frequency Cepstral Coefficients (MFCC) from the second heart sound (S2). The identified S2 is pre-processed with a pre-emphasis filter. It is divided into frames lasting 25 milliseconds and a Hamming window is applied to each frame. The following operations are then performed on each frame: (1) Fast Fourier Transform (FFT) (2) frequencies are linearly spaced into a mel frequency bank, (3) a logarithmic transformation is applied, and (4) a discrete cosine transformation is applied. These steps generate the mel-frequency cepstral coefficients that are used as feature vectors for the training and testing stages.

We used recordings acquired at the 2^nd^ left intercostal space, which is the traditional area for auscultating pulmonary artery and valve events. We identified the approximate location of S2 for each cardiac cycle in a heart sound recording by identifying the loudest signal in a window (composed of 30% of the cardiac cycle) around the T-wave on the simultaneously recorded EKG. (Fig. 1) We searched for features that would estimate the shape of the sound signal spectrum. We took the inverse Fourier transform (IFT) of the logarithm of the estimated spectrum of a signal to create cepstral features. These cepstral features were explored to classify the heart sounds as belonging to a subject with normal or increased pulmonary arterial pressures. Mel-frequency cepstral (MFC) coefficients are applied to extract essential information from a voice signal. They are feature extractors used commonly in audio processing. Mel-frequency cepstral coefficients were extracted by applying the discrete cosine transform to the mel-log powers of the mel-scaling frequency banks. The first 13 MFC coefficients were used in this analysis. Figure 2 shows the steps undertaken to extract the MFC coefficients from each recording.

### Feature Vector Formation

We extracted the features of S2 from every 20 seconds of the heart sound recordings. Each S2 sound was divided into frames by using a 20 millisecond frame length with an overlap of 10 milliseconds. Therefore, successive frames had an overlap equal to 50% of the frame length. Next, a Hamming windowing function was applied to each frame to remove peripheral signals and concentrate the central piece. MFC coefficients were obtained for each frame in the recording by the process described in Fig. 2.

**Figure 3:**
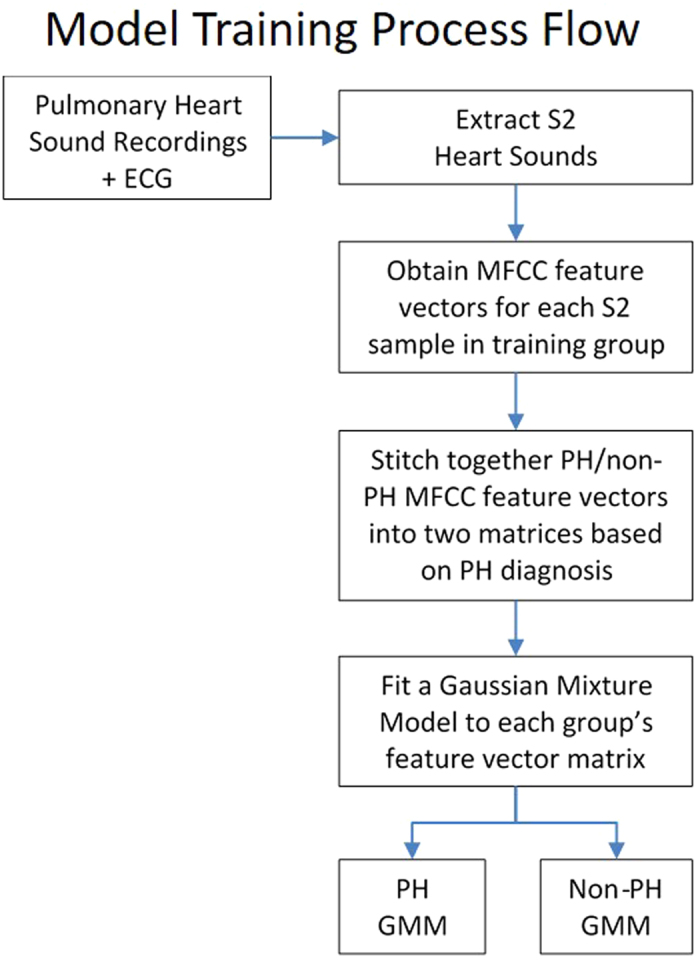
Flow diagram illustrating the training of the acoustic models. The second heart sounds were extracted from the recordings as shown in Fig. 1. The process described in Fig. 2 obtained the Mel-Frequency Cepstral Coefficients (MFCC) feature vectors. The MFCC feature vectors for the subjects with PH (mean PA pressure ≥25 mmHg) were combined into one matrix, and the feature vectors for the subjects with normal PA pressures (mean PA pressure <25 mmHg) were combined into another matrix. A Gaussian Mixture Model (GMM) is fitted to the feature vector matrices, resulting in one model for all subjects with PH and one for all subjects without PH.

The MFC coefficient feature vectors for all PH patients in the training group were then combined into one matrix of size *NxD*, where *N* is the number of frames and *D* is the number of MFC coefficients (13 in our case). Similarly, MFC coefficient feature vectors for all non-PH patients were combined into another matrix. These matrices constituted the training set for each group that were used for training an acoustic model and are described later (Fig. 3).

### Training and Classification

A Gaussian Mixture Model (GMM) classification framework was examined to differentiate between PH and non-PH heart sounds. A GMM models the probability density function (pdf) of the data in each class as a superposition of many Gaussian probability distributions. GMMs can be completely described by three statistical parameters: mean vectors, covariance matrices, and mixture weights. These parameters were estimated for each class by using an Expectation Maximization algorithm.

We ran the analysis and validation steps on several numbers of mixture components. The one that provided the best classification rate determined the number of mixture components of the GMM. Our analysis demonstrated that 8 mixture components gave us the best result. Generally, a Gaussian mixture model that has too few components may not correctly approximate the underlying model, while a mixture with too many components may over-fit the data. We used cross-validation to prevent over-fitting of the data while determining the best number of mixture components to employ. For each class (PH and non-PH), a GMM of 8 Gaussian mixture components was built based on the training set for that group using the MATLAB function *gmdistribution*.*fit*. Classification was performed by obtaining the probability of each class from a given a set of observational subject data. The class that gave the highest probability was assumed to be the class (PH or non-PH) that was most representative of the data, and classified accordingly as either PH or non-PH.

### Classification Procedure

Features of S2 were extracted from the heart sound recording of the patient being tested. They were framed and windowed, and the MFC coefficients were extracted from each frame of each extracted S2. The MFC coefficients were combined into a matrix that was used for testing against each GMM. To classify a patient as PH or non-PH, each frame of the extracted MFC coefficients from the patient was tested against each GMM by obtaining the negative log-likelihood for each GMM with the MATLAB function *posterior*. The likelihood values across all frames for each GMM were averaged to produce a negative log-likelihood value that represented the whole recording. The two final average negative log-likelihoods of the PH and non-PH GMM were compared, and the patient was classified according to the model with the lowest negative log-likelihood.

### Classifier Performance

We evaluated the classification performance using a *k*-fold cross validation with *k* = *5*. A *k*-fold cross validation was performed by randomly dividing the dataset into equal-sized *k* partitions. A single partition was used as the validation data for testing the model, and the remaining *k – 1* partition was used as training data. This was repeated *5* times. Each partition was used once as the validation data. The results from all the iterations were averaged to produce a single estimate of the classification rate. The advantage of *k*-fold cross validation was that all observations were used for training and testing, and each observation was used for validation once. For each set of training and testing groups, the p-value of the mPAp between the testing and training groups was greater than 0.1. This suggested, reassuringly, that the training and testing groups were not statistically different.

The classification rate was obtained for the two groups using the MFC coefficient feature vectors and the GMM classification framework as discussed. We compared the performance of the GMM algorithm with other commonly used machine learning algorithms: K-Nearest Neighbour (KNN), Support Vector Machines (SVM), and ensemble algorithms.

### Comparison with Clinicians

Five physicians with cardiology training, blinded to all clinical data and the patient identity listened to the same164 heart sound recordings and classified the recording as PH or non-PH. The correct rate, false negative rate, and false positive rate were calculated for each clinician.

### Data Presentation and Statistical Analysis

Normally distributed variables are presented as mean ± standard deviation. Non-normally distributed data is presented as a median with a range. P-values for continuous variables were obtained by unpaired unequal variance Student’s t-test for normally distributed variables, and Mann-Whitney U test for non-normally distributed variables. P-values for categorical variables were obtained by chi-squared test of independence. Categorical variables were gender, and WHO classification. All other variables were analyzed as continuous variables. Statistical analysis was performed using the *R programming language environment for statistical computing* (R 3.2.3, 2015, The R Foundation).

Accuracy or correct rate (CR) was calculated as 
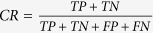
 where *TP* is the number of true positives, *TN* is the number of true negatives, *FP* is the number of false positives, and *FN* is the number of false negatives. The false negative rate (FNR) was calculated as 
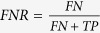
, and the false positive rate (FPR) was calculated as 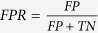.

We generated the receiver-operating-characteristic (ROC) curve by varying the test statistic threshold and calculating the true positive rate and false positive rate for each cut-off point from the likelihood histograms. We plotted the ROC curve as the true positive rate versus the false positive rate.

## Results

The clinical characteristics, diagnosis, phenotype and hemodynamic profiles of the subjects studied are summarized in Tables 1 and 2.

We studied 164 subjects, 78 with mean PA pressures <25 mmHg and 86 subjects with mean PA pressures ≥ 25 mmHg. Subjects with mean PAp ≥ 25 mmHg compared to those with mean PAp < 25 mmHg were older, heavier, with a larger BSA and BMI and reflects the demographics of PH. They also, as would be expected, had a higher WHO or NYHA functional class with more subjects in functional classes II, III and IV. Significantly different and hemodynamically important differences between the 2 groups were found in parameters that reflected pulmonary vascular remodeling and right ventricular dysfunction including mean PAp, mean RAp, transpulmonary gradient (TPG), diastolic pressure gradient (DPG), cardiac index, pulmonary artery capacitance index, pulmonary vascular resistance index.

Minor differences in systemic pressure and resistance index were not hemodynamically significant and again reflect the age and decreased cardiac function found in the subjects with PH.

### Classification Performance and comparison with Physician Performance

**Figure 4:**
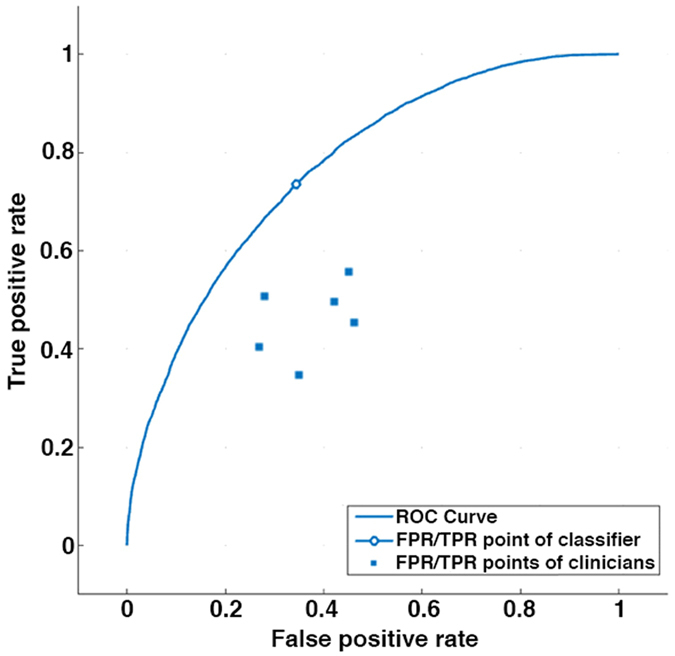
The receiver operating characteristic (ROC) curve for our algorithm to detect the presence or absence of PH. The area-under-the-curve (AUC) was 0.74. The False Positive Rate (FPR)/True Positive Rate (TPR) point for the clinicians’ performance are also shown on the graph. The automated algorithm performs better than clinicians’ interpretation of the recorded heart sounds. X-axis shows the False Positive Rate, and the y-axis shows the True Positive Rate.

The algorithm, that uses 13 MFC coefficients, correctly classified 74% of patients as PH or non-PH. The algorithm resulted in a false negative rate of 23% and a false positive rate of 34%. A receiver operating characteristic (ROC) curve for the algorithm is shown in Fig. 4.

A comparison of the diagnostic accuracy of the the GMM algorithm versus the physicians demonstrated that the GMM algorithm performed with significantly lower false positive rates (34% versus 50% p = 0.04) and significantly lower false negative rates (23% versus 68%, p = 0.0002). The algorithm performed with a significantly better true correct rate of 74% versus 56% compared to the physicians (p = 0.005). Therefore, the speech –recognition-inspired classification algorithm outperformed the clinicians.

### Comparison With Other Machine Learning Algorithms

**Figure 5:**
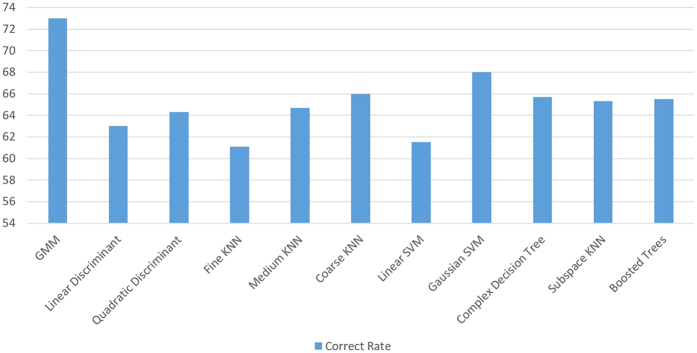
Comparison between the correct rate of the Gaussian Mixture Model (GMM) algorithm and other commonly used machine-learning algorithms. The GMM-based algorithm has a higher correct rate than the other algorithms on our dataset.

Linear discriminant analysis, K-Nearest Neighbour, Support Vector Machines, decision trees, and ensemble algorithms were explored as classifiers to compare their accuracy with the GMM algorithm. Figure 5 illustrates the results of the following machine learning algorithms on our dataset: Linear Discriminant, Quadratic Discriminant, Fine KNN (k = 1), Medium KNN (k = 10), Coarse KNN (k = 100), Linear SVM, Quadratic SVM, Complex Decision Tree, Subspace KNN, and Boosted Trees. The GMM algorithm produced the highest correct rate when compared to alternative algorithms.

## Discussion

The main findings of our study are that our automated machine learning and language- recognition-inspired-speech algorithm diagnosed PH with an accuracy of 74% and outperformed the diagnostic rate of clinicians listening to the same digitized heart sound recordings. In addition, the algorithm performed with lower false negative and false positive rates. We suggest that automated digitally acquired heart sound recordings may be used to screen for PH in the community and prompt referral to specialist centers for echocardiography and cardiac catheterization to reduce the delay in diagnosis and treatment experienced by many patients^3^,^4^,^5^,^16^. The high false positive and negative rates need to be reduced and might be accomplished with an expanded training set or an algorithm that incorporates both our current algorithm and other features that differentiate heart sounds of patients with PH such as reduced entropy^9^.

We did not differentiate between PH and PAH because we are interested in developing a screening tool to diagnose all forms of PH, which could on referral to a specialist center, be evaluated further by echocardiography and cardiac catheterization. However, it is clear from the hemodynamic table that most subjects in the PH group had at least some evidence of pulmonary arterial vascular remodeling based on the diastolic pressure gradient, the transpulmonary gradient and pulmonary arterial capacitance index and pulmonary vascular resistance index. All of these indices have been used to differentiate patients with pulmonary arterial remodeling from patients with PH who have a “passive” increase in mean PAp based on either increased flow, pulmonary arterial wedge pressure, left atrial pressure or left ventricular end diastolic pressure^17^,^18^.

The auscultatory and phonocardiographic indicators of pulmonary hypertension have been described well with plausible biological explanations for the findings^19^,^20^. Indicators of pulmonary hypertension discernable by auscultation include increased loudness of the pulmonary component (P2) of the second heart sound (S2), increased transmission of P2 to the cardiac apex and widening of the interval between the aortic and pulmonary closure sounds^19^,^20^. We have explored previously both the time and frequency domains, as well as, language recognition and machine learning techniques to diagnose pulmonary hypertension in smaller populations of subjects^8^,^9^,^10^.

We used the Zargis Signal X system because when we started our investigations we thought that a simultaneous ECG and pulmonary artery pressure tracing would be a useful adjunct to timing of the second heart sound and ascertaining the relationship with the pulmonary artery pressure tracing. In addition we were unsure of the optimal position for recording the most diagnostic sound information. In previous studies we found that the 2^nd^ left intercostal space and the apex provided superior diagnostic information about pulmonary artery and right ventricular events. However, now that we have developed algorithms for diagnosing pulmonary artery hypertension with and without a simultaneous ECG tracing, we are investigating if we could obtain similar acoustic information with a simple standalone microphone without the extra features of the Zargis Signal X system. This would be an essential next step if we were to apply the algorithm to developing an inexpensive screening device to diagnose PH for use in less privileged or regions remote from medical expertise.

Our analysis uses Mel-frequency cepstral coefficients (MFCC) for extracting features from the heart sounds and Gaussian mixture models (GMM) for classification. MFCC was designed to replicate how the human ear processes sound. This makes it a very good candidate to mimic the sounds clinicians hear when they auscultate patients. GMM is a very mature statistical method that has been used successfully for classifying language, speech, as well as other sounds, with very good classification rates. With enough mixture components, GMMs have the advantage of being able to model probability distributions to high levels of accuracy. They also provide an easy and robust way of fitting the data by using the expectation-maximization (EM) algorithm. The EM algorithm in the context of GMMs is an iterative method that finds the maximum likelihood estimates of the parameters needed to construct a GMM.

Other investigators have studied the use of heart sound spectral analysis to diagnose PH^21^,^22^,^23^,^24^,^25^,^26^. However, these studies either have not included a large human cohort, simultaneous multisite heart sound recording with direct measurement of pulmonary artery pressure or compared the diagnostic accuracy of automated spectral analysis with clinician performance in the diagnosis of pulmonary hypertension. In our analysis, we also compared the performance of the GMM algorithm to other machine learning algorithms that were previously used in the literature to diagnose PH. These algorithms are compared with our GMM algorithm in Fig. 5. On our dataset, the GMM algorithm produces a superior correct rate compared to the other algorithms.

Pulmonary artery catheterization is the gold standard for the diagnosis of PH but is clearly invasive, associated with risk and an unpleasant ordeal for patients^27^,^28^,^29^,^30^. It is unsuitable as a screening tool or for frequent assessment of patient progress or to follow subjects with a family history of PH but who may have normal pulmonary artery pressures for many years. Noninvasive tools such as echocardiography or Magnetic Resonance Imaging (MRI) are either not widely available globally, require expensive machinery, highly qualified personnel to accurately interpret the findings or do not always yield a diagnosis. Doppler echocardiography requires a skillful technician, expert interpretation and may not provide a numerical value of pulmonary artery pressure because of lack of pulmonary or tricuspid insufficiency^31^. The correlation between echocardiographic and direct pulmonary artery pressure measurements in real life situations have been questioned^32^. Magnetic resonance imaging does not provide a numerical value of pulmonary artery pressure but may estimate right ventricular pressure based on imaging of septal position^33^. Magnetic resonance imaging requires a huge capital outlay and is not suited for either continuous or frequent evaluations outside of specialist centers or for use in small or rural community physician’s offices.

There remains a pressing and unmet clinical need for an accurate cost effective screening tool for the early diagnosis of pulmonary hypertension that may be applied by untrained medical personnel to hasten the referral to specialist centers for echocardiography and cardiac catheterization. Our proposed technique is promising, with the results validated against directly and simultaneously measured pulmonary artery pressure. In addition, our technique may outperform clinicians blinded to clinical data. Further work is needed to reduce the false positive and negative rates of diagnosis through refinement of the algorithm, the inclusion of additional features in the spectral analysis, such as the reduced entropy of the first sinusoid formant and make available additional clinical data obtained from the history or EKG to the training set. Future work may incorporate refined ambient noise cancellation technology to improve the quality of digitized heart-sound recordings.

**Figure 6:**
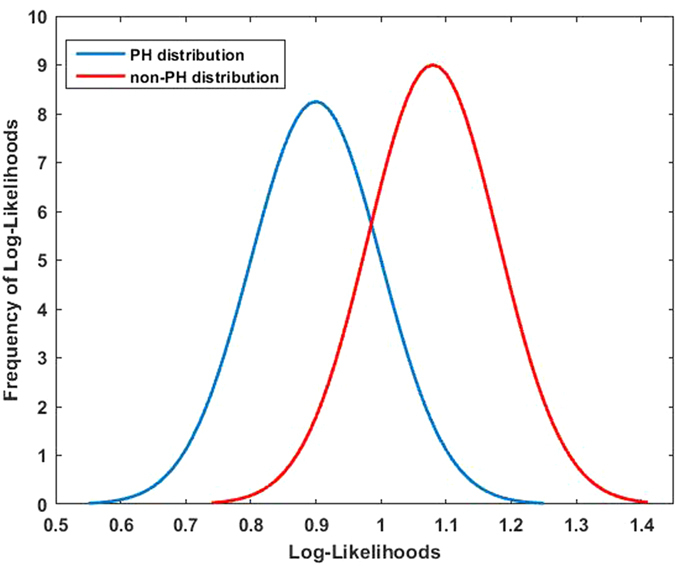
Distribution of negative-log-likelihood ratio between the models. PH group (left blue distribution) and non-PH group (right red distribution). A Gaussian curve is fitted for each distribution and can be seen overlaid on the distributions. The means for the PH and non-PH groups are 0.9 and 1.1 respectively, with a standard deviation of 0.1 for each group. x-axis shows the negative-log-likelihood ratios, and the y-axis shows the frequency.

Clearly the delay in diagnosis of pulmonary hypertension suggests that clinical diagnosis using traditional auscultation is insufficient to meet our patients’ needs, especially if interpreted by non-specialist clinicians as a screening tool. Our analysis is based on a larger dataset than previous studies and includes 164 patients. We performed a power analysis calculation using the log-likelihood as a test statistic. The distribution curves for the PH patients and patients with mPA pressures <25 mmHg are illustrated in Fig. 6. For 95% confidence and 80% statistical power, we would need 9 subjects per group, indicating that the 164 subjects provide more than an adequately powered study from a hypothesis testing perspective. However, clearly larger study sizes will be required for clinical validation in the real world setting. We have described an algorithm that could be incorporated for use with an inexpensive device to record heart sounds using a user-friendly application. The development of this device and application will be the focus of our on going research.

In conclusion, we have shown that there is a direct relationship between the second heart sound and the pulmonary artery pressure. We have developed an automated speech-recognition-machine learning-inspired classification algorithm for the acoustic diagnosis of pulmonary hypertension that outperforms clinicians with lower false positive and negative diagnoses.

## Additional Information

**How to cite this article**: Kaddoura, T. *et al*. Acoustic diagnosis of pulmonary hypertension: automated speech- recognition-inspired classification algorithm outperforms physicians. *Sci. Rep.* **6**, 33182; doi: 10.1038/srep33182 (2016).

## Figures and Tables

**Table 1:** Demographic and Cardiac Catheterization Data of Subjects with a mean PA pressure ≥25 mmHg and <25 mmHg.

	Normal Pulmonary Arterial Pressure (Mean PA pressure <25 mmHg)	Pulmonary Hypertension (Mean PA pressure ≥25 mmHg)	p-value* p < 0.05
Number of Subjects	78	86	
Age (years) median (range)	17 (0.6–86)	39 (0.3–84)	0.04*
Gender	Male = 35	Male = 43	0.6
	Female = 43	Female = 43	
Weight (kg) median (range)	53 (4.4–125)	67 (3.1–168)	0.008
Height (cm) median (range)	155 (49–193)	157 (47–191)	0.05
BSA (m^2^) median (range)	1.5 (0.25–2.6)	1.75 (0.21–2.9)	0.02*
BMI (kg/m^2^) median (range)	21.3 (4.8–38.1)	24.7 (10.3–51.9)	0.02*
NYHA/WHO Classification	Class I (n = 44)	Class I (n = 21)	0.0001*
	Class II (n = 17)	Class II (n = 38)	
	Class III (n = 13)	Class III (n = 15)	
	Class IV (n = 4)	Class IV (n = 12)	
Mean systemic arterial pressure (mmHg); mean ± SD	74 ± 22	82 ± 21	0.02*
Mean right atrial pressure (mmHg); mean ± SD	4 ± 2.5	7 ± 5	1.7*10^−6^*
Mean pulmonary arterial pressure (mmHg); mean ± SD)	17 ± 4	41 ± 13	8.6*10^−31^*
Mean pulmonary arterial wedge pressure (mmHg); mean ± SD	7 ± 4	14 ± 7	3.7*10^−10^*
Transpulmonary gradient (mmHg); mean ± SD	9 ± 4	27 ± 14	1.3*10^−18^*
Diastolic pressure gradient (mmHg); mean ± SD	2 ± 4	12 ± 11	2.6*10^−11^*
Systemic blood flow Index (L/min/m^2^); mean ± SD	3.4 ± 1.2	2.7 ± 0.9	9.1*10^−5^*
Pulmonary blood flow index (L/min/m^2^): mean ± SD	4.1 ± 2	2.9 ± 1.2	2.7*10^−6^*
Heart rate (bpm); mean ± SD	93 ± 23	84 ± 21	0.01*
Pulmonary artery capacitance index (mL/min/m^2^/mmHg); mean ± SD	2.7 ± 1.5	1.1 ± 0.8	3.6*10^−14^*
PVRI (WU.m^2^); mean ± SD	2.6 ± 1.3	11.1 ± 10.5	6.3*10^−11^*
SVRI (WU.m^2^); mean ± SD	23.9 ± 12.5	31.8 ± 16.9	0.0003*
PVR/SVR ratio	0.13 ± 0.08	0.38 ± 0.24	1.3*10^−15^*

bpm = Beats per minute; BMI = Body mass index: BSA = Body surface area; cm = Centimeter; kg = Kilogram; m = Meter; mmHg = Millimeters mercury; mL = milliliter; min = Minute; NYHA = New York Heart Association; WHO = World Health Organization; PA = Pulmonary Artery; PVRI = Pulmonary vascular resistance index; SD = Standard deviation; SVRI = systemic vascular resistance index; WU.m^2^ = Wood units indexed to body surface area; p-value obtained by two-sample unequal variance t-test for continuous variables.

p-value obtained by chi-squared test for categorical variables.

**Table 2:** Associated diagnosis of subjects studied.

Diagnosis	Normal Pulmonary Arterial Pressure Mean PA pressure <25 mmHg N = 78	Pulmonary Hypertension Mean PA pressure ≥25 mmHg N = 86
**Congenital Heart Disease (CHD)**	**n = 38** **Unrepaired CHD n = 31** VSD, s/p PA band n = 1 Anomalous coronary (LCA from RCA) n = 1 ASD prior to device closure n = 12 PDA prior to device closure n = 17 **Repaired CHD n = 7 ** s/p VSD closure n = 3 s/p AVSD repair n = 2 s/p PDA ligation n = 2	**n = 24** **Unrepaired CHD n = 16** ASD n = 12 VSD n = 2 PDA n = 2 **Repaired CHD n = 8** PDA ligation n = 2 AVSD Repair n = 1 VSD Closure n = 1 VSD +COA Repair n = 1 Left pulmonary vein repair n = 3
**Post Heart Transplant**	**n = 24**	**n = 11**
**Idiopathic Pulmonary Arterial Hypertension**	**n = 0**	**n = 16**
**Pre-liver transplant assessment**	**n = 2**	**n = 1**
	**n = 5** Hypertrophic n = 2 Dilated n = 2 Restrictive n = 1	**n = 7** Hypertrophic n = 3 Dilated n = 3 Restrictive n = 1
**Connective Tissue Disease**	**n = 1**	**n = 9**
**Lung Disease**	**n = 7** COPD n = 4 BPD n = 1 ILD n = 2	**n = 11** COPD n = 10 BPD n = 1
**Miscellaneous**	**n = 1** Coronary artery disease	**n = 7** Complete heart block, coronary artery disease s/p coronary bypass surgery n = 1 CTEPH n = 2 Sarcoid n = 1 Pericardial calcification n = 1 Renal failure n = 1 High Altitude Pulmonary Edema n = 1

AVSD = Atrioventricular septal defect; BPD = Bronchopulmonary dysplasia; CHD = Congenital heart disease; COA = Coarctation of the aorta; CTEPH = Chronic thromboembolic pulmonary hypertension; COPD = Chronic obstructive pulmonary disease; ILD = Interstitial lung disease; LCA = Left coronary artery; MR = Mitral regurgitation; mmHg = Millimeter mercury; PA = Pulmonary artery; PDA = Patent ductus arteriosus; RCA = Right coronary artery; S/P = status post; VSD = Ventricular septal defect; WHO = World Health Organization.
